# Trends, prevalence and factors associated with hypertension and diabetes among South African adults living with HIV, 2005–2017

**DOI:** 10.1186/s12889-021-10502-8

**Published:** 2021-03-06

**Authors:** Nicola Chiwandire, Nompumelelo Zungu, Musawenkosi Mabaso, Charles Chasela

**Affiliations:** 1 Division of Epidemiology and Biostatistics, School of Public Health, Faculty of Health Sciences, University of the Witwatersrand, Johannesburg, South Africa; 2 Human Sciences Research Council, Pretoria, South Africa; 3 Department of Psychology, University of Pretoria, Pretoria, South Africa; 4 Implementation Science Unit Programme, Right to Care, Johannesburg, South Africa

**Keywords:** Hypertension, Diabetes, HIV, South Africa, Prevalence, Factors, Trends

## Abstract

**Background:**

Many people are now living longer with HIV due to access to antiretroviral treatment. In turn, there has been an increase in the burden of hypertension and diabetes. The paucity of data on the burden of hypertension and diabetes in adults living with HIV in South Africa is a public health concern. The paper aimed to describe the prevalence and factors associated with hypertension and diabetes among adults living with HIV (ALHIV).

**Methods:**

This was a secondary data analysis of the population based on the South African National HIV Prevalence, Incidence, Behaviour and Communication surveys for 2005, 2008 and 2017. Descriptive statistics were used to summarise the characteristics of the study sample. Bivariate and multivariate logistic regression analyses were used to determine factors associated with hypertension and diabetes.

**Results:**

The total study population of ALHIV aged 25 years and older was 978, 1023 and 2483 for 2005, 2008 and 2017. The prevalence of hypertension showed an increasing trend at 11.8% in 2005, 9.5% in 2008 and 14.3% in 2017. The prevalence of diabetes was 3.3% in 2005, 2.8% in 2008 and 3.2% in 2017. Increased odds of hypertension among adults living with HIV were consistently associated with being female and the age group 45 years older across all the survey years, including pensioners and the sick, living in urban areas, high risk of hazardous alcohol consumption, diabetes and heart disease. Increased odds of diabetes were consistently associated with hypertension across all the survey years, including age group 45 years and older, and poor health. While having a secondary level of education and above was protective against diabetes.

**Conclusion:**

The study showed that the prevalence of hypertension is high and has increased over time among adults living with HIV while the prevalence of diabetes has remained constant. Findings identified factors consistently associated with the prevalence of both diseases overtime, including contemporary risk factors that should be targeted in the integrated management of chronic disease and HIV care model.

**Supplementary Information:**

The online version contains supplementary material available at 10.1186/s12889-021-10502-8.

## Background

At 7.9 million, South Africa has the largest population of people living with HIV (PLHIV) in the world [1, 2]. The successful roll-out of the anti-retroviral therapy (ART) programme since 2004 has contributed to improvements in the life expectancy and viral load suppression in PLHIV; and deaths due to opportunistic infections have also declined [3, 4]. This has transformed HIV infection into a chronic disease and evidence shows that as a consequence of a combination of factors including ageing, being on ART, HIV infection and certain lifestyle choices, their risk of developing non-communicable diseases (NCDs) including hypertension or diabetes increases [5]. In addition, the growing double burden of the HIV and NCD co-epidemics has put significant pressure not only on the affected individuals but also on the struggling health system, existing health programmes and the economy in South Africa [6–10].

Studies that have been done to characterise the burden of hypertension and diabetes in PLHIV highlight the implication of not only collecting prevalence estimates but also adequately addressing multimorbidity in PLHIV and ensuring adequate availability of treatment to reduce the risk [7]. However, these studies present varied findings, suggesting that results and conclusions may not necessarily be extrapolated from one country to the next and not one intervention fits all [11–18]. A meta-analysis and systematic review across sub-Saharan Africa reported prevalence estimates for hypertension in PLHIV ranging between 5.2 and 50.0% and that of diabetes to range between 0.5 and 36.6%. In South Africa, studies have also reported prevalence estimates in PLHIV for hypertension as high as 38.6% and diabetes as high as 8.66% [19, 20]. However, these estimates cannot be generalised to the entire South African population. Common risk factors of these NCDs in PLHIV have also been reported to include ageing, family history, use of ART, HIV infection, urbanisation, alcohol drinking and high body mass index in studies conducted in Ethiopia, Nigeria, Malawi, Tanzania, Zimbabwe and South Africa [13, 21–27]. Also, further studies indicated that hypertension and diabetes can also act as risk factors of each other [12].

Improved understanding of the magnitude of hypertension and diabetes and associated factors among PLHIV in South Africa is vital for informing public health interventions in the country. This paper investigates trends, prevalence and associated factors of hypertension and diabetes among adults living with HIV (ALHIV) in South Africa using the 2005, 2008, 2012, and 2017 nationally representative population-based HIV Prevalence, Incidence, Behaviour and Communication Surveys.

## Methodology

### Study design and sample

This secondary analysis utilised data collected from the South African National HIV Prevalence, Incidence, Behaviour, and Communication (SABSSM) surveys in 2005, 2008 and 2017. This was a multi-stage stratified cluster randomised cross-sectional population-based survey undertaken by the Human Sciences Research Council (HSRC). The details of the study and sampling method are described in detail elsewhere [1, 28–30]. Briefly, in the 2005 and 2008 surveys, in each household a maximum of three people were selected randomly to participate in the study, each representing the 2–14 years, 15–24 years and 25 years and older age groups [28, 29]. In the 2012 and 2017 surveys, all household members were eligible to participate in the survey [1, 30]. The analysis for this paper was restricted to ALHIV aged 25 years and older who responded to the questions on the primary outcomes, hypertension and diabetes.

### Study variables

The primary outcome variables were hypertension and diabetes. These variables were self-reported by the survey participants when asked the question “Do you currently have any of the following illnesses?”. Both outcome variables were coded 1 for the answer “yes”, and 0 for the answer “no”. The explanatory variables used in this study were grouped into three categories namely socio-demographic, behavioural and health-related factors. The socio-demographic factors included sex (male and female), age (25–34 yrs., 35–44 yrs. and 45+ yrs.), race (Non- Black African including White, Coloured and Indian; and Black African), marital status (never married and ever married), employment status (unemployed, employed and other (incl. Old age pensioner, sick/disabled and unable to work), educational level (no or up to primary and secondary and above), locality type (urban, urban formal, urban informal, tribal area/rural informal and rural formal) and province. The behavioural factors included hazardous alcohol consumption using the Alcohol Use Disorders Identification Test score (no risk (do not drink alcohol), low risk (0–7), medium risk (8–15), high risk (16–19) and addiction likely (20–40). Hazardous alcohol consumption was defined as a habit or amount of alcohol intake that raises the risk of adverse health effects [31]. The health-related factors included the exposure to ARVs (ARV exposed, ARV naive), viral load suppressed, perception of general health (excellent, good, fair, and poor), cancer, mental distress, tuberculosis, heart disease, diabetes and health care access (public care, and private care).

### Statistical analysis

All analyses were adjusted for complex survey design, clustering and any bias due to non-response using sample weights and the *svyset* and *svy* commands in STATA version 15 [32]. Frequency tables with percentages and median with interquartile ranges (IQR) were used to describe the characteristics of the study sample and trends in the prevalence of hypertension and diabetes in the study population. Pearson’s chi-squared test was used for comparison of categorical variables. Bivariate logistic regression analysis was done and explanatory variables that had a *p*-value < 0.25 were included in the multivariate logistic regression model to determine the associated factors. The Wald test was used to keep variables that had a *p*-value < 0.05 through a process of removing, refitting, and verifying until all significant variables were kept in the final main effects model. Variables which had very wide confidence intervals were removed from the model regardless of the *p*-value. The Variance Inflation Factor test was done to check for multicollinearity at a cut off level of 5. A post-estimation test using the Hosmer-Lemeshow Goodness of Fit (GOF) test was done to test whether the final model was a good fit [33]. If the GOF test failed multiple times, the preliminary final model was used instead. Crude and adjusted odds ratios (aOR), 95% confidence intervals (CI) and *p*-values ≤0.05 are reported to indicate the level of significance.

## Results

### Trends in the prevalence of hypertension and diabetes

A total of 978, 1023 and 2483 study participants aged 25 years and older were included for 2005, 2008 and 2017 respectively. Of these, the overall prevalence of hypertension was 11.8% in 2005, 9.5% in 2008 and 14.3% in 2017. While the overall prevalence of diabetes was 3.3% in 2005, 2.8% in 2008 and 3.2% in 2017 (Tables 1 and 2). There was an increase in the trend of hypertension over the 3 years, whereas that of diabetes remained stagnant. The median age (IQR) for those who were hypertensive increased over the period from 37 (33–47) years in 2005, to 44 (34–50) years in 2008 and 48 (38–55) years in 2017 (Table 1). The median age for diabetes also increased from 35 (29–44) years in 2005 to 47 (37–53) years in 2008 and 49 (42–57) years in 2017. Additionally, there was a decreased trend in hypertension and diabetes in the 25–34 age group over the 3 years, while the trend in the 45+ years age group increased.

**Table 1:** Sociodemographic characteristics and hypertension among ALHIV 25 years and older by survey year for 2005, 2008 and 2017 in South Africa

	2005			2008			2017		
Population characteristics	Hypertension	No hypertension	***p***-value	Hypertension	No hypertension	***p***-value	Hypertension	No hypertension	***p***-value
Overall	**126 (11.8%)**	**820 (88.2%)**		**107 (9.5%)**	**859 (90.5%)**		**372 (14.3%)**	**1955 (85.7%)**	
Sex									
Female	97 (13.9%)	542 (86.1%)	0.057	93 (12.8%)	578 (87.2%)	< 0.001	289 (16.6%)	1365 (83.4%)	< 0.001
Male	29 (8.2%)	278 (91.8%)		14 (3.0%)	281 (97.0%)		83 (10.1%)	590 (89.9%)	
Age									
Median (IQR)	37 (33–47)	34 (29–39)		44 (34–50)	34 (29–39)		48 (38–55)	36 (31–44)	
25–34 years	30 (8.6%)	396 (91.4%)	0.009	21 (4.9%)	396 (95.1%)	< 0.001	48 (5.8%)	761 (94.3%)	< 0.001
35–44 years	37 (12.0%)	288 (88.0%)		31 (7.9%)	296 (92.1%)		84 (9.8%)	647 (90.2%)	
45+ years	59 (21.3%)	136 (78.7%)		55 (26.1%)	167 (73.7%)		240 (30.6%)	547 (69.4%)	
Race									
Non-Black African	15 (10.4%)	65 (89.6%)	0.757	4 (13.9%)	54 (86.1%)	0.476	41 (20.3%)	153 (79.7%)	0.087
Black African	111 (11.8%)	754 (88.2%)		103 (9.4%)	805 (90.6%)		331 (14.1%)	1802 (85.9%)	
Marital status									
Never married	41 (9.5%)	437 (90.5%)	0.121	54 (8.5%)	524 (91.6%)	0.154	192 (11.9%)	1288 (88.1%)	< 0.001
Ever married	84 (14.3%)	380 (85.7%)		53 (11.7%)	332 (88.3%)		180 (18.9%)	667 (81.1%)	
Highest educational qualification									
None or Primary	68 (15.1%)	315 (84.9%)	0.147	56 (13.4%)	274 (86.6%)	0.072	101 (18.9%)	441 (81.1%)	0.002
Secondary and above	58 (9.74%)	503 (90.3%)		50 (8.0%)	580 (92.0%)		199 (12.3%)	1253 (87.7%)	
Employment status									
Unemployed	59 (9.9%)	462 (90.1%)	0.108	54 (9.9%)	423 (90.1%)	0.314	249 (15.4%)	1199 (84.6%)	< 0.001
Employed	40 (13.0%)	291 (87.0%)		34 (7.9%)	345 (92.1%)		99 (11.0%)	718 (89.0%)	
Other	26 (20.3%)	64 (79.7%)		19 (13.4%)	90 (86.6%)		23 (33.7%)	37 (66.3%)	
Locality									
Urban formal	57 (12.7%)	326 (87.3%)	0.491	47 (12.4%)	323 (87.6%)	0.072	236 (15.9%)	1027 (84.1%)	0.008
Urban informal	34 (14.2%)	180 (85.8%)		30 (10.3%)	208 (89.7%)				
Tribal area/Rural informal	28 (10.7%)	239 (89.3%)		25 (6.1%)	238 (93.9%)		99 (11.6%)	636 (88.4%)	
Rural formal	7 (6.2%)	75 (93.8%)		5 (5.9%)	90 (94.1%)		37 (10.1%)	292 (89.9%)	
Hazardous alcohol consumption									
No risk	88 (13.2%)	558 (86.8%)	0.085	73 (9.7%)	574 (90.3%)		261 (14.1%)	1359 (85.9%)	
Low risk	24 (6.7%)	175 (93.3%)		25 (11.4%)	181 (88.6%)	0.230	83 (16.8%)	407 (83.2%)	0.078
Medium risk	12 (16.4%)	52 (83.6%)		6 (5.2%)	62 (94.8%)		13 (7.1%)	137 (92.9%)	
High risk	1 (5.5%)	11 (94.5%)		1 (1.2%)	15 (98.8%)		8 (22.6%)	23 (77.4%)	
Addiction likely	1 (4.4%)	11 (95.6%)		1 (1.7%)	12 (98.3%)		4 (9.3%)	25 (90.7%)	
Exposure to ARVs									
ARV exposed							234 (15.1%)	1150 (84.9%)	0.478
ARV naive							107 (13.0%)	600 (87.0%)	
Viral load suppressed									
Yes							254 (15.9%)	1231 (84.1%)	0.033
No							116 (11.9%)	703 (88.1%)	
Perception of general health									
Excellent	5 (7.3%)	88 (92.7%)	0.003	5 (2.2%)	161 (97.8%)	0.006	42 (10.0%)	371 (90.0%)	< 0.001
Good	55 (9.0%)	498 (91.0%)		48 (9.9%)	444 (90.1%)		186 (13.2%)	1134 (86.8%)	
Fair	50 (19.3%)	182 (80.7%)		43 (14.5%)	194 (85.5%)		105 (18.4%)	370 (81.6%)	
Poor	15 (22.9%)	51 (77.1%)		11 (10.0%)	59 (90.0%)		39 (27.1%)	78 (72.9%)	
Diabetes									
Yes	20 (56.1%)	14 (43.9%)	< 0.001	17 (54.0%)	10 (46.0%)	< 0.001	49 (52.7%)	35 (47.3%)	< 0.001
No	103 (10.3%)	804 (89.7%)		88 (7.7%)	849 (92.3%)		322 (13.1%)	1919 (86.9%)	
Heart disease									
Yes				7 (28.8%)	12 (71.2%)	0.008	23 (41.8%)	25 (58.2%)	< 0.001
No				99 (8.7%)	845 (91.3%)		346 (13.6%)	1922 (86.4%)	
Cancer									
Yes	3 (54.5%)	2 (45.5%)	0.009	3 (65.5%)	3 (34.5%)	< 0.001	4 (66.2%)	4 (33.8%)	< 0.001
No	120 (11.5%)	816 (88.5%)		102 (8.8%)	855 (91.2%)		365 (14.2%)	1946 (85.8%)	
Mental distress									
Yes	71 (18.2%)	327 (81.8%)	0.004	5 (8.6%)	31 (91.4%)	0.951	128 (16.1%)	512 (83.9%)	0.230
No	53 (7.52%)	491 (92.5%)		99 (8.9%)	826 (91.1%)		244 (13.7%)	1443 (86.3%)	
Tuberculosis									
Yes	9 (15.4%)	54 (84.6%)	0.467	5 (7.6%)	44 (92.5%)	0.757	16 (19.5%)	60 (80.5%)	0.257
No	114 (11.5%)	766 (88.5%)		100 (9.0%)	815 (91.0%)		352 (14.1%)	1891 (85.9%)	
Health care access									
Public	109 (12.7%)	675 (87.3%)	0.113	90 (10.6%)	649 (89.4%)	0.104	337 (14.7%)	1766 (85.3%)	0.296
Private	15 (7.1%)	122 (92.9%)		15 (6.3%)	186 (93.7%)		35 (12.7%)	164 (87.3%)

**Table 2:** Sociodemographic characteristics and diabetes among ALHIV 25 years and older by survey year for 2005, 2008 and 2017 in South Africa

	2005			2008			2017		
Population characteristics	Diabetes	No diabetes	***p***-value	Diabetes	No diabetes	***p***-value	Diabetes	No diabetes	***p***-value
Overall	**34 (3.3%)**	**907 (96.7%)**		**29 (2.8%)**	**937 (97.2%)**		**87 (3.2%)**	**2242 (96.8%)**	
Sex									
Female	22 (2.5%)	614 (97.5%)	0.198	26 (3.5%)	645 (96.5%)	0.227	63 (3.6%)	1592 (96.4%)	0.208
Male	12 (4.5%)	293 (95.5%)		3 (1.5%)	292 (98.5%)		24 (2.5%)	650 (97.5%)	
Age									
Median (IQR)	35 (29–44)	34 (29–40)		47 (37–53)	34 (29–40)		49 (42–57)	37 (32–46)	
25–34 years	10 (3.1%)	415 (96.9%)	0.866	5 (1.1%)	411 (98.9%)	< 0.001	4 (0.7%)	804 (99.3%)	< 0.001
35–44 years	12 (3.1%)	312 (96.9%)		9 (2.2%)	317 (97.8%)		21 (2.8%)	710 (97.2%)	
45+ years	12 (4.1%)	180 (95.9%)		15 (9.2%)	209 (90.8%)		62 (6.8%)	728 (93.2%)	
Race									
Non-Black African	3 (0.9%)	76 (99.1%)	0.030	3 (8.0%)	55 (92.0%)	0.103	10 (5.6%)	183 (94.4%)	0.184
Black African	31 (3.3%)	830 (96.7%)		26 (2.7%)	882 (97.3%)		77 (3.1%)	2059 (96.9%)	
Marital status									
Never married	11 (3.2%)	465 (96.8%)	0.944	11 (2.0%)	566 (98.0%)	0.097	38 (2.8%)	1441 (97.2%)	0.250
Ever married	23 (3.3%)	438 (96.7%)		18 (4.5%)	368 (95.5%)		49 (3.9%)	801 (96.1%)	
Highest educational qualification									
None or Primary	12 (3.0%)	370 (97.0%)	0.915	12 (4.5%)	318 (95.5%)	0.157	34 (7.1%)	509 (92.9%)	< 0.001
Secondary and above	22 (3.5%)	535 (96.5%)		16 (2.2%)	614 (97.9%)		37 (1.7%)	1416 (98.3%)	
Employment status									
Unemployed	17 (3.0%)	501 (97.0%)	0.915	17 (2.4%)	460 (97.6%)	0.528	56 (3.4%)	1394 (96.6%)	0.582
Employed	13 (3.7%)	317 (96.3%)		9 (2.9%)	370 (97.1%)		29 (3.0%)	788 (97.0%)	
Other (incl. Old age pensioner. Sick/disabled and unable to work)	4 (3.4%)	85 (96.6%)		3 (5.0%)	106 (95.0%)		2 (1.4%)	58 (98.6%)	
Locality									
Urban formal	18 (4.2%)	361 (95.8%)	0.271	11 (3.1%)	359 (96.9%)	0.797	56 (3.5%)	1209 (96.5%)	0.241
Urban informal	10 (4.8%)	203 (95.2%)		6 (2.0%)	231 (98.0%)				
Tribal area/Rural informal	6 (2.1%)	261 (97.9%)		9 (2.5%)	255 (97.53%)		24 (2.8%)	711 (97.2%)	
Rural formal	0 (0.0%)	82 (100%)		3 (4.6%)	92 (95.4%)		7 (1.4%)	322 (98.6%)	
Hazardous alcohol consumption									
No risk	22 (3.4%)	623 (96.7%)	0.040	22 (3.2%)	625 (96.8%)	0.679	62 (3.2%)	1559 (96.9%)	0.548
Low risk	5 (1.1%)	191 (98.9%)		5 (1.9%)	201 (98.1)		19 (3.0%)	472 (97.0%)	
Medium risk	6 (9.6%)	57 (90.4%)		0 (0.0%)	68 (100%)		4 (5.3%)	146 (94.7%)	
High risk	0 (0.0%)	12 (100%)		1 (2.4%)	15 (97.6%)		1 (0.2%)	30 (99.8%)	
Addiction likely	1 (6.0%)	11 (94.0%)		0 (0.0%)	13 (100%)		0 (0.0%)	29 (100%)	
Exposure to ARVs									
ARV exposed							56 (3.2%)	1331 (96.8%)	0.675
ARV naive							26 (3.3%)	680 (96.7%)	
Viral load suppressed									
Yes							56 (3.0%)	1430 (97.0%)	0.392
No							30 (3.5%)	790 (96.5%)	
Perception of general health									
Excellent	1 (1.5%)	92 (98.5%)	0.001	1 (0.9%)	165 (99.1%)	0.031	6 (1.2%)	406 (98.8%)	< 0.001
Good	14 (1.8%)	537 (98.2%)		10 (2.0%)	481 (98.0%)		42 (2.6%)	1278 (97.5%)	
Fair	15 (8.4%)	214 (91.6%)		15 (6.2%)	223 (93.8%)		22 (4.1%)	457 (95.9%)	
Poor	4 (3.9%)	62 (96.1%)		3 (2.7%)	67 (97.3%)		17 (13.9%)	99 (86.1%)	
Hypertension									
Yes	20 (15.6%)	103 (84.4%)	< 0.001	17 (16.1%)	88 (83.9%)	< 0.001	49 (11.3%)	322 (88.7%)	< 0.001
No	14 (1.6%)	804 (98.4%)		10 (1.4%)	849 (98.7%)		35 (1.7%)	1919 (98.3%)	
Heart disease									
Yes				5 (22.1%)	15 (77.9%)	< 0.001	4 (5.5%)	44 (94.5%)	0.298
No				23 (2.4%)	920 (97.6%)		83 (3.1%)	2188 (96.9%)	
Cancer									
Yes	3 (54.5%)	2 (45.5%)	< 0.001	2 (48.7%)	4 (51.3%)	< 0.001	3 (41.5%)	5 (58.5%)	< 0.001
No	31 (2.9%)	905 (97.1%)		25 (2.3%)	933 (97.7%)		84 (3.1%)	2230 (96.9%)	
Mental distress									
Yes	19 (5.0%)	376 (95.0%)	0.061	3 (8.9%)	33 (91.1%)	0.053	25 (3.4%)	615 (96.6%)	0.789
No	15 (2.1%)	527 (97.9%)		24 (2.3%)	902 (97.7%)		62 (3.1%)	1627 (96.9%)	
Tuberculosis									
Yes	4 (7.4%)	57 (92.6%)	0.134	4 (7.1%)	45 (92.9%)	0.116	8 (5.9%)	68 (94.1%)	0.142
No	30 (3.0%)	850 (97.0%)		24 (2.5%)	892 (95.5%)		79 (3.1%)	2168 (96.9%)	
Health care access									
Public	28 (3.2%)	753 (96.8%)	0.559	21 (2.3%)	719 (97.7%)	0.190	69 (2.8%)	2034 (97.2%)	0.002
Private	6 (4.4%)	130 (95.6%)		7 (5.1%)	193 (94.9%)		17 (8.1%)	184 (91.9%)	

In 2005, 2008 and 2017 females had a higher prevalence of hypertension at 13.9, 12.8 and 16.6% respectively compared to males. Black Africans had the highest prevalence of hypertension at 11.8% in 2005 whereas, non-black Africans had the highest prevalence in 2008 and 2017 with 13.9 and 20.3% respectively. The prevalence estimates for diabetes were as follows: Black Africans with 3.3% in 2005, Non-Black Africans with 8.0% in 2008 and Non-Black Africans with 5.6% in 2017. In 2017, those who were exposed to ARVs had a high prevalence of hypertension at 15.1% and a marginally lower prevalence of diabetes at 3.2% compared to those who were naïve to ARVs (Tables 1 and 2).

**Fig. 1:**
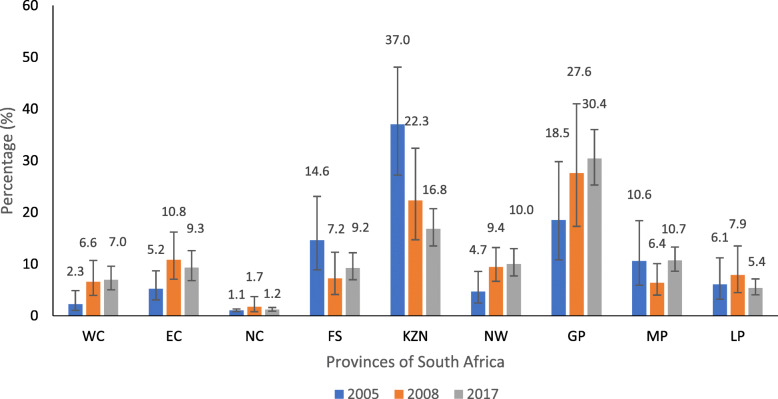
Prevalence of hypertension among ALHIV by provinces in 2005, 2008 & 2017

The prevalence distribution of hypertension was highest in the KwaZulu Natal province at 37.0% in 2005 and for 2008 and 2017, the Gauteng province had the highest proportion of 27.6 and 30.4% respectively (Fig. 1).

**Fig. 2:**
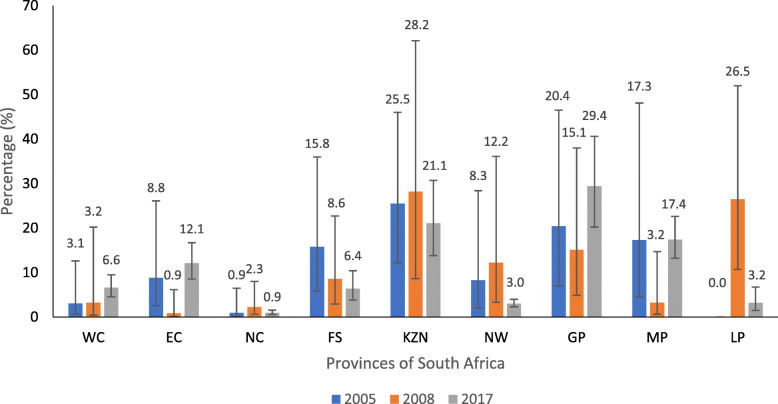
Prevalence of diabetes among ALHIV by provinces in 2005, 2008 & 2017

The illustration shows that diabetes was highest in the KwaZulu Natal province in 2005 and 2008 at 25.5 and 28.2% respectively, and in 2017, the Gauteng Province had the highest estimation at 29.4% (Fig. 2).

### Factors associated with hypertension

The factors associated with hypertension in 2005 included being female (aOR = 2.59; 95% CI = 1.26 to 5.32), the age group 45+ years (aOR = 4.03; 95% CI = 1.96 to 8.28), those with diabetes (aOR = 11.73; 95% CI = 4.02 to 34.20), and those with mental distress (aOR = 2.97; 95% CI = 1.59 to 5.54) (Table 3). In 2008, factors associated with hypertension included being female (aOR = 4.31; 95% CI = 1.94 to 9.58), the age group 45+ years (aOR = 8.44; 95% CI = 3.92 to 18.15), those who perceived their health to be good (aOR = 3.72; 95% CI = 1.00 to 13.81), those who perceived their health to be fair (aOR = 5.44; 95% CI = 1.40 to 21.12), those who perceived their health to be poor (aOR = 4.82; 95% CI = 1.05 to 22.20), those who lived in an urban formal area (aOR = 2.95; 95% CI = 1.34 to 6.53), those with diabetes (aOR = 6.16; 95% CI = 1.68 to 22.59) and those who accessed public health care services (aOR = 2.58; 95% CI = 1.02 to 6.51) (Table 4). While in 2017 factors associated with hypertension included being female (aOR = 2.33; 95% CI = 1.60 to 3.42), the age group 45+ years (aOR = 7.32; 95% CI = 4.78 to 11.21), pensioners and the sick (aOR = 2.27; 95% CI = 1.09 to 4.73), those who lived in an urban area (aOR = 1.61; 95% CI = 1.16 to 2.23), those who had a high risk of hazardous alcohol consumption (aOR = 4.43; 95% CI = 1.67 to 11.76), those with diabetes (aOR = 5.17; 95% CI = 2.69 to 9.96) and those with heart disease (aOR = 3.36; 95% CI = 1.59 to 7.10) (Table 5).

**Table 3:** Factors associated with hypertension for 2005

Variables in the model	Crude OR (95% CI)	Adjusted OR (95% CI)
Sex		
Male	Reference	Reference
Female	1.81 (0.98–3.37)	2.59 (1.26–5.32) *
Age groups		
25–34 years	Reference	Reference
35–44 years	1.46 (0.73–2.92)	1.72 (0.81–3.64)
45+ years	2.90 (1.43–5.89) **	4.03 (1.96–8.28) ***
Race		
Non-Black African	Reference	Reference
Black African	1.15 (0.47–2.85)	0.96 (0.34–2.81)
Hazardous alcohol consumption		
No risk	Reference	Reference
Low risk	0.47 (0.23–0.95) *	0.56 (0.26–1.24)
Medium risk	1.28 (0.55–2.98)	1.48 (0.60–3.69)
High risk	0.38 (0.05–3.18)	0.38 (0.04–3.37)
Addiction likely	0.29 (0.03–2.44)	0.18 (0.01–2.92)
Diabetes		
No	Reference	Reference
Yes	11.16 (4.14–30.05) ***	11.73 (4.02–34.20) ***
Mental distress		
No	Reference	Reference
Yes	2.73 (1.54–4.85) **	2.97 (1.59–5.54) **

*p < 0.05, **p < 0.01, ***p < 0.001

**Table 4:** Factors associated with hypertension for 2008

Variables in the model	Crude OR (95% CI)	Adjusted OR (95% CI)
Sex		
Male	Reference	Reference
Female	4.81 (2.10–11.02) ***	4.31 (1.94–9.58) ***
Age groups		
25–34 years	Reference	Reference
35–44 years	1.68 (0.76–3.71)	2.22 (0.99–4.97)
45+ years	6.95 (3.34–14.45) ***	8.44 (3.92–18.15) ***
Race		
Non-African	Reference	Reference
African	0.65 (0.19–2.17)	0.67 (0.14–3.28)
Locality		
Tribal area/Rural informal	Reference	Reference
Rural formal	0.96 (0.25–3.66)	0.93 (0.35–2.46)
Urban informal	1.77 (0.90–3.51)	2.40 (1.06–5.43) *
Urban formal	2.20 (1.13–4.28) *	2.95 (1.34–6.53) **
Hazardous alcohol consumption		
No risk	Reference	Reference
Low risk	1.20 (0.61–2.36)	1.44 (0.66–3.12)
Medium risk	0.50 (.018–1.42)	0.78 (0.26–2.35)
High risk	0.11 (0.01–0.97) *	0.15 (0.01–1.78)
Addiction likely	0.16 (0.02–1.47)	0.25 (0.03–1.97)
Perception of general health		
Excellent	Reference	Reference
Good	4.92 (1.49–16.22) **	3.72 (1.00–13.81) *
Fair	7.56 (2.33–24.58) **	5.44 (1.40–21.12) *
Poor	4.95 (1.30–18.80) *	4.82 (1.05–22.20) *
Diabetes		
No	Reference	Reference
Yes	14.03 (5.27–37.35) ***	6.16 (1.68–22.59) **
Heart disease		
No	Reference	Reference
Yes	4.26 (1.33–13.70) *	1.13 (0.23–5.52)
Health care access		
Private	Reference	Reference
Public	1.76 (0.88–3.51)	2.58 (1.02–6.51) *

*p < 0.05, **p < 0.01, ***p < 0.001

**Table 5:** Factors associated with hypertension for 2017

Variables in the model	Crude OR (95% CI)	Adjusted OR (95% CI)
Sex		
Male	Reference	Reference
Female	1.76 (1.29–2.41) ***	2.33 (1.60–3.42) ***
Age groups		
25–34 years	Reference	Reference
35–44 years	1.78 (1.11–2.83) *	1.83 (1.12–3.00) *
45+ years	7.24 (4.88–10.74) ***	7.32 (4.78–11.21) ***
Race		
Non-Black African	Reference	Reference
Black African	0.65 (0.39–1.07)	0.61 (0.33–1.13)
Employment status		
Unemployed	Reference	Reference
Employed	0.68 (0.48–0.98) *	0.76 (0.50–1.15)
Other (incl. Old age pensioner, sick/disabled and unable to work)	2.80 (1.45–5.40) **	2.27 (1.09–4.73) *
Locality		
Rural informal	Reference	Reference
Rural (farms)	0.85 (0.51–1.42)	1.58 (0.86–2.90)
Urban	1.44 (1.08–1.91) *	1.61 (1.16–2.23) **
Exposure to ARVs		
ARV naive	Reference	Reference
ARV exposed	1.20 (0.85–1.68)	0.97 (0.67–1.40)
Hazardous alcohol consumption		
No risk	Reference	Reference
Low risk	1.23 (0.88–1.71)	1.26 (0.83–1.93)
Medium risk	0.47 (0.24–0.91) *	0.61 (0.29–1.30)
High risk	1.78 (0.57–5.57)	4.43 (1.67–11.76) **
Addiction likely	0.78 (0.31–1.94)	1.10 (0.43–2.78)
Diabetes		
No	Reference	Reference
Yes	7.38 (4.41–12.35) ***	5.17 (2.69–9.96) ***
Heart disease		
No	Reference	Reference
Yes	4.57 (2.35–8.88) ***	3.36 (1.59–7.10) **

*p < 0.05, **p < 0.01, ***p < 0.001

### Factors associated with diabetes

The factors associated with diabetes in 2005 included being Black African (aOR = 5.70; 95% CI = 1.10 to 29.44) and those with hypertension (aOR = 11.71; 95% CI = 3.78 to 36.29) (Table 6). In 2008, factors associated with diabetes included the age group 45+ years (aOR = 4.44; 95% CI = 1.00 to 19.73), those who had hypertension (aOR = 7.56; 95% CI = 2.29 to 24.92) and those who had heart disease (aOR = 6.92; 95% CI = 1.62 to 29.62) (Table 7). While in 2017 factors associated with diabetes included the age group 45+ years (aOR = 7.90; 95% CI = 2.11 to 29.58), those who reported having poor health (aOR = 6.48; 95% CI = 1.65 to 25.41), those who had hypertension (aOR = 4.60; 95% CI = 2.34 to 9.07), those who had secondary and above education (aOR = 0.31; 95% CI = 0.16 to 0.59) and those who accessed public health care (aOR = 0.25; 95% CI = 0.11 to 0.60) (Table 8).

**Table 6:** Factors associated with diabetes for 2005

Variables in the model	Crude OR (95% CI)	Adjusted OR (95% CI)
Sex		
Male	Reference	Reference
Female	0.55 (0.22–1.39)	0.41 (0.14–1.20)
Age groups		
25–34 years	Reference	Reference
35–44 years	1.00 (0.35–2.89)	0.88 (0.29–2.66)
45+ years	1.32 (0.44–3.93)	0.58 (0.19–7.75)
Race		
Non-Black African	Reference	Reference
Black African	3.77 (1.04–13.65) *	5.70 (1.10–29.44) *
Perception of general health		
Excellent	Reference	Reference
Good	1.20 (0.15–9.78)	1.41 (0.26–7.62)
Fair	5.91 (0.71–48.97)	5.16 (0.92–29.01)
Poor	2.61 (0.25–27.00)	2.30 (0.33–16.05)
Hypertension		
No	Reference	Reference
Yes	11.16 (4.14–30.05) ***	11.71 (3.78–36.29) ***
Mental distress		
No	Reference	Reference
Yes	2.47 (0.93–6.54)	1.09 (0.40–2.92)
Health facility of choice		
Private	Reference	Reference
Public	0.73 (0.25–2.14)	0.42 (0.15–1.11)

*p < 0.05, **p < 0.01, ***p < 0.001

**Table 7:** Factors associated with diabetes for 2008

Variables in the model	Crude OR (95% CI)	Adjusted OR (95% CI)
Sex		
Male	Reference	Reference
Female	2.31 (0.57–9.32)	1.28 (0.28–5.77)
Age groups		
25–34 years	Reference	Reference
35–44 years	2.03 (0.46–8.98)	1.95 (0.43–8.86)
45+ years	9.14 (2.67–31.27) **	4.44 (1.00–19.73) *
Race		
Non-African	Reference	Reference
African	0.32 (0.08–1.35)	0.39 (0.09–1.81)
Hypertension		
No	Reference	Reference
Yes	14.03 (5.27–37.35) ***	7.56 (2.29–24.92) ***
Heart disease		
No	Reference	Reference
Yes	11.71 (3.02–45.39) ***	6.92 (1.62–29.62) **

*p < 0.05, **p < 0.01, ***p < 0.001

**Table 8:** Factors associated with diabetes for 2017

Variables in the model	Crude OR (95% CI)	Adjusted OR (95% CI)
Sex		
Male	Reference	Reference
Female	1.47 (0.80–2.70)	1.80 (0.86–3.78)
Age groups		
25–34 years	Reference	Reference
35–44 years	3.88 (1.01–14.93) *	6.38 (1.80–22.62) **
45+ years	9.88 (2.96–32.95) ***	7.90 (2.11–29.58) **
Race		
Non-African	Reference	Reference
African	0.54 (0.211–1.36)	0.52 (0.15–1.82)
Educational level		
None or Primary	Reference	Reference
Secondary and above	0.22 (0.13–0.39) ***	0.31 (0.16–0.59) ***
Exposure to ARVs		
ARV naive	Reference	Reference
ARV exposed	0.98 (0.54–1.77)	0.94 (0.47–1.85)
Perception of general health		
Excellent	Reference	Reference
Good	2.18 (0.86–5.52)	1.15 (0.41–3.26)
Fair	3.56 (1.30–9.81) *	1.04 (0.32–3.35)
Poor	13.46 (4.63–39.15) ***	6.48 (1.65–25.41) **
Hypertension		
No	Reference	Reference
Yes	7.38 (4.41–12.35) ***	4.60 (2.34–9.07) ***
Heart disease		
No	Reference	Reference
Yes	1.79 (0.59–5.46)	0.77 (0.18–3.31)
Healthcare access		
Private	Reference	Reference
Public	0.34 (0.17–0.70) **	0.25 (0.11–0.60) **

*p < 0.05, **p < 0.01, ***p < 0.001

## Discussion

This study found an overall prevalence of hypertension among ALHIV in 2017 of 14.3%, and this was an increase since 2005. This hypertension prevalence is comparable to other African studies that also report estimates ranging between 10.2 and 17.4% [12, 18, 34–36]. On the other hand, the overall prevalence of diabetes was 3.2% in 2017, which remained the same as in 2005 and 2008. This prevalence is lower than previously reported in other African countries with estimates ranging between 5.6 and 8.66% [12–14, 20, 36, 37]. This may have been attributed to the participants not being aware of diabetes-related symptoms leading to the observed underestimation and this is supported by an IDF report stating that Africa has the highest proportion of undiagnosed diabetes [38]. Nevertheless, as PLHIV are more likely to interact with health facilities where hypertension screening is routine, case-finding and linkage to treatment can occur [39]. However different measures for diabetes will need to be implemented as routine vital checks at health facilities do not typically include diabetes tests unless patients have been recently diagnosed and appear “unwell” [39]. The findings also show that in 2005 most hypertensive and diabetic ALHIV were from the KwaZulu-Natal province. However, for 2017, the Gauteng province had the highest proportions of hypertensive and diabetic participants. Comparative studies to determine possible reasons for the shift from 2005 to 2017 could not be found at the time of this study. Nonetheless, according to the 2005 SABSSM survey, the KwaZulu-Natal province had a significantly higher number of PLHIV than the other provinces thus supporting our findings [28]. The shift in 2017 may have been due to urbanisation, migration and a rise in the elderly population which may be attributed to ART in PLHIV [40].

Our study also found that females, aged 45 years and older, and those who resided in an urban area had the highest prevalence of both NCDs for all 3 years. The high prevalence in women may be as a result of an underestimation in men reflecting more on their health-seeking behaviour than disease prevalence itself [14, 18, 34, 35, 41, 42]. The impact of urbanisation has also been documented in studies such as the 2012 SANHANES study and the South African cross-sectional study on HIV positive educators which suggest that urban living leads to easy access to unhealthy diets and a sedentary lifestyle [34, 35]. In 2008 and 2017, we further see that those who had primary education and below also had a high prevalence of hypertension and diabetes as supported by Ntuli et al. [43]. Wang et al. suggest that not only an unhealthy diet but even a lack of regular and effective physical exercise and higher alcohol intake can be correlated with low levels of schooling [44]. The presence of comorbidities was also evident with higher proportions of individuals with cancer, heart disease, TB and mental distress reporting having hypertension and diabetes. This is consistent with studies which observed similar findings globally and in Africa [34, 45–47]. This could mean that PLHIV who have a comorbidity require close monitoring to ensure that their health conditions are effectively managed thereby reducing the risk of acquiring more NCDs.

Factors that increased the odds of hypertension for all 3 years in our study included being female, aged 45 years and older, or having diabetes. Antonello et al., Malaza et al., and Zungu et al. suggest that increased odds in females were mainly attributed to women having higher BMI’s and hip to weight ratios than men and women being more physically inactive, all of which are considered risk factors [35, 41, 48]. Ageing has also been well described to result in gradual vascular stiffening and the prolonged use of ART particularly protease inhibitors is related to the production of vascular reactive oxygen species resulting in hypertension [17, 23, 24]. While diabetes has been suggested by Petrie et al., and Gazzaruso et al. to result from hypertension-linked insulin resistance due to multiple pathophysiological mechanisms influencing microvascular and endothelial functioning [24, 25]. Mental distress also increased the odds of having high blood pressure in 2005. An American study found that one’s mental health affects one’s ability to maintain a healthy lifestyle, seek early treatment for comorbid conditions, or consistently adhere to treatment programs [45]. Living in an urban area was another significant factor associated with hypertension in 2008 and 2017. Findings from previous studies suggest that urbanisation and the associated lifestyle change including poor diet and low physical activity are risk factors of hypertension [49]. While having a high risk of hazardous alcohol consumption or having heart disease were also additional associated factors for hypertension found in 2017. In Zimbabwean and Brazilian studies alcohol consumption increased the risk of hypertension, however, there have been several hypotheses proposed for this association [50, 51]. Some researchers have to an extent been able to characterize the pathogenesis due to harmful alcohol drinking, citing that a reduction in vasodilators like nitric oxide occurs leading to an inflammatory lack of relaxation and oxidative injury of the blood vessel lining [52]. Other researchers have suggested the link between harmful alcohol consumption and the eventual accumulation of triglycerides and total cholesterol, both of which have been documented to have a link to hypertension [53]. In addition, according to the WHO, heart disease can result from hypertension in the general population [54]. Hypertension and diabetes have long been documented to be a major modifiable risk factor for heart disease due to the progressive damage to the heart that occurs over time [55, 56]. Lastly, in 2017, the employment category “other” which included pensioners, sick and disabled people, was also associated with hypertension. The reasoning for this includes that pensioners are presumably older hence the effect of age may have attributed to this finding; also, sick and disabled people are more likely to be non-mobile and be physically inactive both of which again are characteristics that have been documented to increase the risk of hypertension.

The common significant factor associated with diabetes across the years was having hypertension and the supporting reasons and studies are identical to those mentioned above. In 2008 other additional factors included being 45 years and above and having heart disease; both established risk factors in other studies [49]. In 2017, being 45 years and above, and reporting poor health was significantly associated with diabetes in the study population. Also, having secondary and above education level lowered one’s odds of having diabetes. Other studies with a similar finding reported that education increased awareness and therefore the individuals were more likely to make better health-related decisions [18]. The above findings from our study, thus, offer various avenues for possible interventions which may include active awareness campaigns targeted at men, those who have educational qualifications below secondary level or those residing in urban areas. Also, as the HIV population is ageing, closely monitoring the impact of antiretroviral medication could be considered. Lastly, these findings could allow for the refinement of the integrated chronic care model allowing for increased case finding.

### Limitations

This study had a few limitations. Due to the cross-sectional nature of the surveys, causality cannot be determined in the study population. Questionnaire data used were self-reported and therefore prone to social desirability, recall bias and underreporting. The analysis did not account for unmeasured and unreported risk factors and other confounders that may have had an impact on the outcome variables. Also, as hypertension and diabetes were not measured or diagnostically determined, misclassification bias may have been introduced. Lastly, wide confidence intervals for some variables were observed and this may have been as a result of a small sample answering particular questions, therefore, conclusions drawn from the data need to be replicated with a larger sample size. Nevertheless, the surveys were nationally representative and can be generalised to adults aged 25 years and above living with HIV with hypertension and diabetes.

## Conclusion

Population-based nationally representative surveys are important for developing sound health decisions and policies that would affect the country. This study identified factors consistently associated with the prevalence of hypertension and diabetes among ALHIV overtime. Additional contemporary risk factors were also identified. Therefore, these findings contribute to understanding the trend and identifying modifiable factors responsible for hypertension and diabetes among ALHIV adults from 2005 to 2017. This may help inform the development of integrated chronic disease management model to reduce the co-morbid burden of disease and associated adverse health outcomes.

## Supplementary Information

**Additional file 1: Table S1.** Characteristics of adults living with HIV in South Africa: 2005, 2008 & 2017. **Figure S1.** Distribution of study participants by province in 2005, 2008 & 2017.

## Data Availability

The dataset(s) are accessible on request on the HSRC data research repository http://datacuration.hsrc.ac.za/.

## Abbreviations

ALHIV: Adults living with HIV
ART: Antiretroviral Therapy
AUDIT: Alcohol Use Disorder Identification Test
CDC: Centers for Disease Control and Prevention
CI: Confidence Interval
HIV: Human immunodeficiency virus
HSRC: Human Sciences Research Council
IDF: International Diabetes Federation
NCDs: Non-communicable diseases
OR: Odds ratios
PLHIV: People Living with HIV
REC: Research Ethics Committee
SABSSM: South African National HIV Prevalence, Incidence, Behaviour and Communication survey
SANHANES: South African National Health and Nutrition Examination Survey
WHO: World Health Organization
