# Supplementary material for: Trends, prevalence and factors associated with hypertension and diabetes among South African adults living with HIV, 2005–2017
Source: BMC Public Health. 2021 Mar 6;21:462. doi: 10.1186/s12889-021-10502-8 (PMC7937211; doi:10.1186/s12889-021-10502-8)
Supplement: Supplementary file 1 — Additional file 1: Table S1. Characteristics of adults living with HIV in South Africa: 2005, 2008 & 2017. Figure S1. Distribution of study participants by province in 2005, 2008 & 2017. [file 12889_2021_10502_MOESM1_ESM.docx]

Trends, prevalence and factors associated with hypertension and diabetes among South African adults living with HIV, 2005-2017

**Nicola Chiwandire^1^*, Nompumelelo Zungu^2,3^, Musawenkosi Mabaso^2^, Charles Chasela^1,4^**

* [nvchiwandire@gmail.com](mailto:nvchiwandire@gmail.com)

Supplementary Table 1. Characteristics of adults living with HIV in South Africa: 2005, 2008 and 2017

|  | 2005 | | 2008 | | 2017 | |
| --- | --- | --- | --- | --- | --- | --- |
| Overall | **n (N=978)** | **%** | **n (N=1023)** | **%** | **n (N=2483)** | **%** |
| Sex |  |  |  |  |  |  |
| Female | 658 | 61.1% | 705 | 66.1% | 1762 | 65.3% |
| Male | 320 | 38.9% | 318 | 33.9% | 721 | 34.7% |
| Age |  |  |  |  |  |  |
| Median (IQR) | 34 (29 - 41) |  | 34 (29 - 40) |  | 38 (32 - 46) |  |
| 25 – 34 years | 436 | 50.3% | 441 | 50.8% | 852 | 37.6% |
| 35 – 44 years | 338 | 32.6% | 344 | 31.6% | 789 | 33.1% |
| 45+ years | 204 | 17.1% | 164 | 17.6% | 842 | 29.3% |
| Race |  |  |  |  |  |  |
| Non-Black African | 84 | 2.5% | 64 | 2.0% | 218 | 3.6% |
| Black African | 893 | 97.4% | 959 | 98.0% | 2265 | 96.4% |
| Marital status |  |  |  |  |  |  |
| Never married | 488 | 50.4% | 581 | 62.9% | 1485 | 62.2% |
| Ever married | 473 | 46.5% | 395 | 33.3% | 852 | 33.1% |
| Educational level |  |  |  |  |  |  |
| None or Primary | 387 | 37.3% | 336 | 27.4% | 544 | 18.6% |
| Secondary and above | 575 | 59.8% | 637 | 68.8% | 1459 | 64.6% |
| Employment status |  |  |  |  |  |  |
| Unemployed | 533 | 56.2% | 483 | 52.9% | 1456 | 59.8% |
| Employed | 334 | 32.3% | 385 | 34.5% | 818 | 33.1% |
| Other (incl. old age pensioner. sick/disabled and unable to work) | 94 | 8.4% | 110 | 9.1% | 61 | 2.4% |
| Locality |  |  |  |  |  |  |
| Urban formal | 400 | 43.8% | 387 | 42.5% | 1340 | 64.6% |
| Urban informal | 222 | 14.9% | 267 | 18.4% |  |  |
| Tribal area/Rural informal | 274 | 35.4% | 272 | 32.4% | 781 | 30.1% |
| Rural formal | 82 | 5.9% | 97 | 6.6% | 362 | 5.3% |
| Hazardous alcohol consumption |  |  |  |  |  |  |
| No risk | 659 | 68.3% | 655 | 68.1% | 1627 | 64.8% |
| Low risk | 202 | 19.5% | 208 | 17.6% | 491 | 21.3% |
| Medium risk | 65 | 6.3% | 69 | 6.2% | 150 | 6.5% |
| High risk | 13 | 1.1% | 16 | 1.3% | 31 | 1.5% |
| Addiction likely | 13 | 1.2% | 13 | 1.5% | 30 | 1.1% |
| Exposure to ARVs |  |  |  |  |  |  |
| ARV exposed |  |  |  |  | 1471 | 62.1% |
| ARV naive |  |  |  |  | 742 | 33.3% |
| Viral load suppressed |  |  |  |  |  |  |
| Yes |  |  |  |  | 1582 | 63.2% |
| No |  |  |  |  | 874 | 35.6% |
| Perception of general health | |  |  |  |  |  |
| Excellent | 95 | 9.9% | 167 | 18.3% | 415 | 17.3% |
| Good | 560 | 61.5% | 497 | 48.2% | 1322 | 54.3% |
| Fair | 240 | 20.5% | 241 | 22.6% | 479 | 18.8% |
| Poor | 68 | 5.2% | 71 | 7.2% | 117 | 4.8% |
| Diabetes |  |  |  |  |  |  |
| Yes | 34 | 3.2% | 29 | 2.7% | 87 | 3.0% |
| No | 907 | 93.2% | 937 | 92.6% | 2242 | 92.0% |
| Hypertension |  |  |  |  |  |  |
| Yes | 126 | 11.4% | 107 | 9.1% | 372 | 13.6% |
| No | 820 | 85.2% | 859 | 86.5% | 1955 | 81.3% |
| Heart disease |  |  |  |  |  |  |
| Yes |  |  | 20 | 1.8% | 48 | 2.4% |
| No |  |  | 946 | 93.2% | 2272 | 92.4% |
| Cancer |  |  |  |  |  |  |
| Yes | 5 | 0.6% | 6 | 0.4% | 8 | 0.3% |
| No | 939 | 96.0% | 959 | 94.7% | 2315 | 94.5% |
| Mental distress |  |  |  |  |  |  |
| Yes | 552 | 58.2% | 36 | 3.3% | 789 | 31.6% |
| No | 409 | 38.9% | 928 | 91.6% | 1694 | 68.4% |
| Tuberculosis |  |  |  |  |  |  |
| Yes | 67 | 7.1% | 50 | 4.8% | 76 | 3.5% |
| No | 881 | 89.7% | 917 | 90.5% | 2247 | 91.4% |
| Health care access |  |  |  |  |  |  |
| Public | 800 | 82.8% | 749 | 74.4% | 2109 | 85.8% |
| Private | 139 | 11.8% | 202 | 18.9% | 210 | 8.8% |


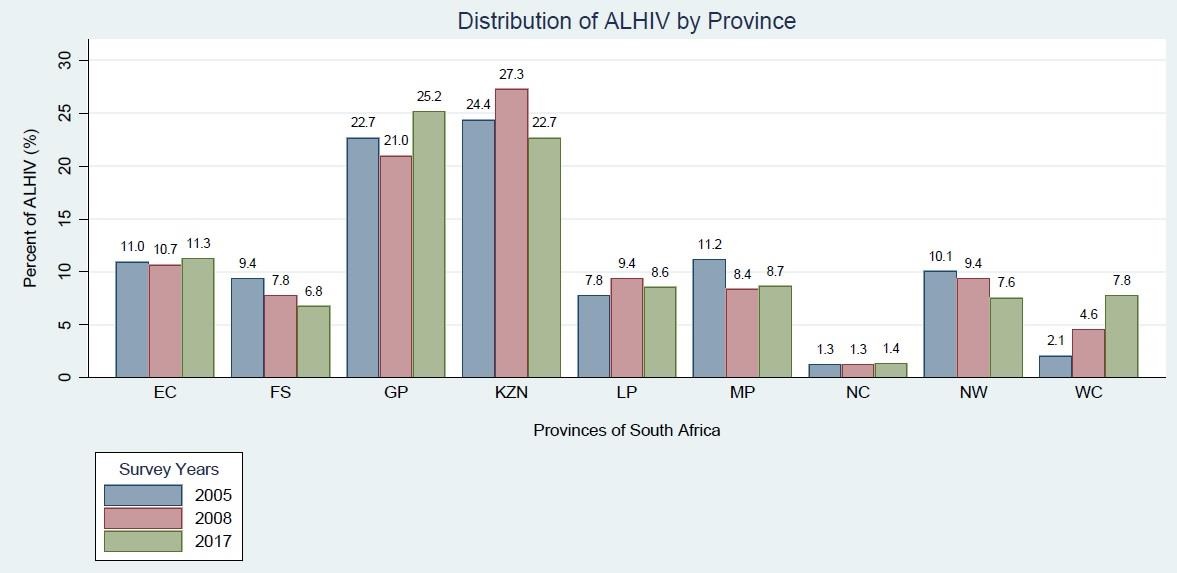


Supplementary Figure 1: Distribution of study participants by province in 2005, 2008 & 2017
